# Motor vehicle accident is a risk factor for traumatic head injury among children in Abuja: analysis of the first trauma registry in Nigeria

**DOI:** 10.11604/pamj.2019.33.215.19289

**Published:** 2019-07-16

**Authors:** Abdul Rahman Shour, Benjamin Holmes, Emmanuel Adoyi Ameh, Oluwole Olayemi Olaomi, Ronald Anguzu, Laura Dawn Cassidy

**Affiliations:** 1 Institute for Health and Equity, Medical College of Wisconsin, Milwaukee, Wisconsin, USA; 2 National Hospital Abuja, Abuja, Federal Capital Territory, Nigeria

**Keywords:** Pediatric trauma, mechanism of injury, motor vehicle accident, head injuries, lower and middle-income countries, Nigeria trauma registry

## Abstract

**Introduction:**

Pediatric traumatic injury is a major public health concern that is poorly documented in lower and middle-income countries. This study analyzed data on pediatric injuries from a unique hospital trauma registry in Abuja, Nigeria.

**Methods:**

Data were analyzed on 220 traumatically injured patients aged 21 years/less to describe injury characteristics and to determine the association between mechanism of injury and pediatric head injuries in Abuja, Nigeria, between 2014 and 2015. Bivariate analysis using Pearson's chi-square and adjusted logistic regression were conducted to characterize the population and identify risk factors for head injury. P-values<0.05 were considered statistically significant. All statistical analyses were performed using STATA v.15.1.

**Results:**

The majority of patients were male (60.9%) with a mean age (SD) of 12.5±6.9 years. Head injuries were most common (49.6%), followed by chest (14.1%), abdomen (12.3%) and back (7.7%). The mechanism of injury was statistically significantly associated with head injury (p=0.027) with 63% of children in a motor vehicle accident sustaining a head injury. After adjusting for covariates, the odds of head injury were 3.8 times higher for children injured in a motor vehicle accidents (MVA) compared to those with falls (95%CI 1.40-10.40).

**Conclusion:**

This analysis reveals that motor vehicle accident is a risk factor for traumatic head injury among children in Nigeria. Therefore, efforts should be made to address motor vehicle accidents involving children. These data will help to inform age-related prevention and treatment strategies. The results of this study highlight the importance of collecting pediatric trauma data in developing countries.

## Introduction

A traumatic injury refers to a wound or physical injury that requires immediate medical care [1, 2]. The injury episode could be intentional (assault, suicide, homicide) or unintentional (fall, drowning, car crash) and could result in severe fractures, traumatic brain injury or loss of consciousness and Post-Traumatic Stress Disorders (PTSD) [1, 2]. Injury-related deaths increased by nearly 10.7% worldwide, from 4.3 million deaths in 1990 to 4.8 million in 2013 [3] and are expected to increase by nearly 28% by the year 2030 [4]. Between 1990 and 2010, unintentional injuries accounted for approximately 12% of injury-related deaths globally among children/adolescents aged 1-19 years [5, 6]. The burden of trauma-related morbidity and mortality is increasing in Lower and Middle-Income Countries (LMICs) [7]. Pediatric traumatic injury is responsible for the highest mortality in children and adolescents in LMICs [8-11]. Traumatic injury is an understudied neglected epidemic in LMICs, particularly in areas where precautionary measures are often missing and health-care systems lack standardized data to guide improvement strategies [6, 12-14]. The cost of these injuries places a severe burden on already stressed healthcare infrastructures and the economies of LMICs. The economic burden of injury in LMICs is significant. Studies have projected that injury cost per disability-adjusted life years is high [15]. Pediatric trauma has become a significant source of morbidity and mortality in sub-Saharan Africa (SSA) [16, 17]. More than 50% of all global injuries occurred in SSA, with 68.0 deaths per 100000 population, compared to 6.4 in Western Europe [16, 17]. This regional inequity between high and low-income countries is driven by the unequal distribution of wealth and risks, which undermines human productivity and opportunities for preventing pediatric unintentional injuries [5, 18]. The investment disparity between less developed and developed countries remains significant and this mirrors the insufficient allocation of healthcare resources [19] and the low priority given to injury prevention in LMICs [20]. In the United States trauma registries are part of an integrated trauma system and provide essential data to guide interventions, reduce mortality, improve care and guide policy [21-25]. However, there are very few existing trauma registries in LMICs. For the few trauma registries that exit, poor data quality remains a challenge.

In Nigeria, pediatric traumatic injury is a major public health concern [8, 11, 26]. Nigeria is one of the poorest countries in the world with 61.2% of its population living below $1 USD per day [27, 28]. The injury prevalence in adult and children is 11.2 per 100,000 population [24]. More male children are affected by injuries than females [29]. Pediatric mortality in Nigeria has been reported to occur mainly from severe head injuries, abdominal and chest injury [16] and include injuries resulting from drowning, burns, assault and falls [5, 7, 16, 29, 30]. Previous studies have examined severe head injuries [31-33], childhood trauma [17] and injury-related deaths [16, 25] in Nigeria [17, 23]. However, due to poor data quality, these studies used limited categories of variables and were mainly descriptive data analysis [7, 17]. Additionally, these studies did not examine the risk factors of head injuries and correlates among Nigeria’s pediatric population. There is a need to understand the predictors of head injuries among children in order to develop targeted interventions to decrease the burden on the Nigerian healthcare system. While prior research has identified differences in injury-related deaths due to severe head trauma, little literature exists regarding other correlates of pediatric head trauma in Nigeria. This study analyzed potential risk factors for pediatric head injuries and correlates of these head injuries, using the first hospital trauma registry in Abuja, Nigeria, between the years 2014 and 2015.

## Methods

Data and sample: trauma registry data on pediatric injuries treated at Abuja National Hospital were analyzed. Details of the trauma registry development have been previously described [23]. Data were collected using the Research Electronic Data Capture (REDCap), a secured, web-based application for data collection and management [23]. De-identified data on 220 traumatically injured pediatric patients aged 21 years or less were analyzed to describe injury characteristics, for the years 2014 and 2015. The study was approved as exempt by the Institutional Review Boards of Abuja National Hospital and the Medical College of Wisconsin. First, a descriptive analysis was conducted to characterize the population. Then an analysis of head injuries was conducted because it is the leading cause of death from injury in children. Covariates were defined as: gender (male or female), age categories (0-7, 8-14, 15-21), injury location (road/street, home/school, missing), brought in by (Relative, friend, police, other, missing), season (wet and dry), Glasgow Coma Scale (GCS) (severe (3-8), moderate (9-12), mild (13-15), missing), chest injury (no, yes, missing), back injury (no, yes, missing), injury to the abdomen (no, yes, missing) and survival (no, yes, missing). Data were analyzed to compare the characteristics between children with a head injury and those with other injuries using an unadjusted/bivariate analysis and Pearson's chi-square test. A multiple logistic regression analysis was used to model the relationship between head injuries and associated risk factors adjusting for covariates. P-values<0.05 were considered statistically significant and all statistical analyses were performed using STATA v.15.1 [34].

## Results

As shown in Table 1, over half of the injured patients were male (60.9%) and 29.6% were between ages 0-7, 22.7% were between ages 8-14 years and 47.7% were between ages 15 and 21 years. The majority of injuries (80.9%) occurred on the street (either as pedestrian or motor vehicle accident) and 7.7% occurred in the home or school settings. Overall, 67.7% of injured patients were brought to the hospital by their relatives, 13.1% by their friend, 4.6% by the police, and 10.5% by ambulance or other people. More accidents took place during the wet season compared to the dry season (55.5% vs 44.5%), respectively. Motor vehicle accidents were a most common mechanism of injury (46.8%), followed by assault (15.9%), domestic accidents (15.0%), falls (10.9%) and burns (10.0%). In terms of body regions, head injuries were most common (49.6%), followed by chest (14.1%), abdomen (12.3%) and back (7.7%). Based on GCS, 86.3% of injured patients suffered a mild injury, 4.6% a moderate injury and 4.6% a severe injury. Of those with discharge status recorded, 2.7% of patients died, 68.7% survived and 28.6% were unknown due to missing values.

**Table 1:** Patient demographics (N=220)

	n (%)
**Gender**	
Male	134 (60.9)
Female	86 (39.1)
**Age category**	
0-7	65 (29.6)
8-14	50 (22.7)
15-21	105 (47.7)
**Injury Location**	
Road/street	178 (80.9)
Home/school	17 (7.7)
Missing	25 (11.4)
**Brought in by**	
Relative	149 (67.7)
Friend	29 (13.1)
Police	10 (4.6)
Other	23 (10.5)
Missing	9 (4.1)
**Season**	
Wet	122 (55.5)
Dry	98 (44.5)
**Mechanism of Injury**	
Burns	22 (10.0)
Falls	24 (10.9)
Domestic Accidents	33 (15.0)
Motor Vehicle Accidents (MVA)	103 (46.8)
Assault	35 (15.9)
Missing	3 (1.4)
**Glasgow Coma Scale (GCS)**	
Severe (3-8)	10 (4.6)
Moderate (9-12)	10 (4.6)
Mild (13-15)	109 (86.3)
Missing	10 (4.5)
**Chest injury**	
No	170 (77.3)
Yes	31 (14.1)
Missing	19 (8.6)
**Back injury**	
No	182 (82.7)
Yes	17 (7.7)
Missing	21 (9.6)
**Injury to the Abdomen**	
No	173 (78.6)
Yes	27 (12.3)
Missing	20 (9.1)
**Head injury**	
No	97 (44.1)
Yes	109 (49.6)
Missing	14 (6.3)
**Transportation to Hospital**	
No (walk)	132 (60.0)
Yes (hospital, relative)	75 (34.1)
Missing	13 (5.9)
**Survival**	
No	6 (2.7)
Yes	151 (68.7)
Missing	63 (28.6)

**Factors associated with head injuries:** as shown in Table 2, the mechanism of injury was statistically significantly associated with head injury (p=0.027) with 63% of children injured in motor vehicle accident sustaining a head injury. The odds of head injury were 3.8 times higher for children injured in motor vehicle accident compared to those injured in falls (95% CI 1.40-10.40). Compared to those with falls, the risks of head injuries were 3.1 times higher for children with burns (95% CI 0.84-11.06), 1.9 times for children injured in a domestic accident (95% CI 0.58-6.04) and 1.9 times higher for children who experienced assault (95% CI 0.56-6.05), although not statistically significant (Table 3).

**Table 2:** Unadjusted bivariate analysis using chi-squares (outcome=head injuries)

	Children with No Head injuries, n=97 n (%)	Children with Head Injuries, n=109 n (%)	p-value
**Gender**			
Male	59 (46.8)	67 (53.2)	0.925
Female	38 (47.5)	42 (52.5)	
**Age category**			
0-7	32 (53.3)	28 (46.7)	0.227
8-14	25 (52.1)	23 (47.9)	
15-21	40 (40.8)	58 (59.2)	
**Brought in by**			
Relative	66 (46.8)	75 (53.2)	0.190
Friend	13 (44.8)	16 (55.2)	
Police	2 (20)	8 (80)	
Other	14 (60.9)	9 (39.1)	
**Season**			
Wet	57 (49.6)	58 (50.4)	0.423
Dry	40 (43.9)	51 (56.1)	
**Mechanism of Injury (MOI)**			
MVA	37 (37.0)	63 (63.0)	0.027
Falls	17 (70.8)	7 (29.2)	
Domestic Accidents	16 (57.1)	12 (42.9)	
Burns	8 (44.4)	10 (55.6)	
Assault	18 (51.4)	17 (48.6)	
**Chest injury**			
No	83 (48.8)	87 (51.2)	0.458
Yes	12 (41.4)	17 (58.6)	
**Back injury**			
No	86 (47.3)	96 (52.7)	0.988
Yes	8 (47.1)	9 (52.9)	
**Injury to the Abdomen**			
No	80 (46.2)	93 (53.8)	0.367
Yes	15 (55.6)	12 (44.4)	
**Survival**			
No	1 (20.0)	4 (80.0)	0.202
Yes	73 (48.9)	76 (51.1)	

**Table 3:** Results of logistic regression analysis (Outcome=Head injuries)

	Odds Ratio	95% CI
**Gender**		
Male (ref)		
Female	1.12	0.62 – 2.04
**Age category**		
0-7 (ref)		
8-14	0.90	0.40 – 2.02
15-21	1.43	0.68 – 3.00
**Mechanism of Injury**		
Falls (ref)		
Burns	3.05	0.84 – 11.06
Domestic Accident	1.87	0.58 – 6.04
MVA	3.82	1.40 – 10.40
Assault	1.85	0.56 – 6.05

## Discussion

This study examined risk factors of pediatric head injuries and correlates of these head injuries, using the first hospital trauma registry in Abuja, Nigeria, between 2014 and 2015. This analysis reveals that motor vehicle accident is a risk factor for traumatic head injury among children in Nigeria. Injuries were more prevalent in males than in females. This study demonstrates the feasibility and needs to maintain a trauma registry in an LMIC hospital. Among children treated at a hospital in Abuja, Nigeria, most were injured in a car crash and head injuries were the most common injury. The results of this study highlight the importance of collecting pediatric trauma data in developing countries. Previous studies have examined severe head trauma in childhood [17, 31-33] and injury-related deaths [16, 25] in Nigeria's pediatric population, mainly using descriptive data analysis [17, 23]. However, these studies [16, 17, 25, 31-33] did not examine risk factors of pediatric head injuries using a national trauma registry and were limited to a subset of variable categories that have been shown to drive head injuries in other populations such as pre- and school-aged children [35]. Our results are consistent with previous studies finding that motor vehicle accidents are responsible for a significant percentage of head injuries among children in Nigeria [29, 32, 36, 37], Ghana, Uganda and Tanzania [38-43]. This analysis adds to the literature by providing information on the risk factors for traumatic head injury among Nigerian children.

For several decades, head injuries in children remain a persistent public health problem resulting from motor vehicle accidents [31, 44]. By utilizing potential covariates to explain the odds of pediatric head injuries in Nigeria, this study also contributes to the literature by helping to understand how best to target future interventions to address the burden of head trauma on children and families. Our findings that injuries were more prevalent in males than females, and children between ages 15 and 21 years, underscore several public health and policy implications. These data will help to inform age-related prevention and treatment strategies. With increased importation, number and use of motor vehicles, there is a need for enhanced enforcement of road safety strategies such as speed bumps, seat belt use and yielding to traffic laws in SSA. Enforcing the WHO road safety recommendations to improve national road safety in LMIC should also be considered [45-47]. More attention must be given to the needs of pedestrians and cyclists, child restraint use, emergency care and the need to strengthen the enforcement of road safety legislation [47]. The effect of injuries to a child, especially a severe head injury, impacts the family and their socioeconomic status, access to health care and associated financial burden [48].

The results of this study illustrate the importance of standardized ongoing data collection and analyses to identify patterns of injury, risk factors and associated outcomes [49]. Due to paucity of routinely collected, standardized injury data in LMICs, improving prevention, injury surveillance and outcomes can prove challenging [7]. As this is the first trauma registry in Nigeria, there were limitations to the data. Data were only included on children who made it to the hospital to be treated. There were missing data on mortality as many patients left against medical advice or their deaths may not have been captured in the registry. However, these data take the first important step to demonstrate the importance of continued efforts to routinely collect data and continuous quality improvement efforts to address pediatric traumatic injury in Nigeria. Finally, addressing the above limitations would provide good opportunities for future research including the use of mixed methods approaches.

## Conclusion

This analysis reveals that motor vehicle accident is a risk factor for traumatic head injury among children in Nigeria. Injuries were more prevalent in males than females, and children between ages 15 and 21 years are more vulnerable compared to other age groups. Therefore, efforts should be made to address motor vehicle accidents involving children. These data will help to inform age-related prevention and treatment strategies. The results of this study highlight the importance of collecting pediatric trauma data in developing countries.

### What is known about this topic

- Pediatric traumatic injury is a major public health concern that is poorly documented in lower and middle-income countries;
- Sub-Saharan Africa accounts for more than 50% of all global injuries, with 68.0 deaths per 100000 population, compared to 6.4 in Western Europe;
- Previous studies have examined severe head trauma in childhood and injury-related deaths in Nigeria's pediatric population, but did not examine risk factors of pediatric head injuries using a national trauma registry and were limited to a subset of variable categories that have been shown to drive head injuries.

### What this study adds

- This analysis reveals that motor vehicle accident is a risk factor for traumatic head injury among children in Nigeria;
- Among children treated at a hospital in Abuja, Nigeria, most were injured in a car crash and head injuries were the most common injury;
- This study demonstrates the feasibility and needs to maintain a trauma registry in a low- and middle-income hospital and the results of this study highlight the importance of collecting pediatric trauma data in developing countries.

## Competing interests

The authors declare no competing interests.
